# Supplementary material for: CAR T cell targeting of inflammatory myeloid progenitors in the bone marrow remodels border-associated macrophages and reverses cognitive aging
Source: bioRxiv. 2026 Sep 28:2026.09.27.754777. Preprint. [Version 1] doi: 10.64898/2026.09.27.754777 (PMC13648559; doi:10.64898/2026.09.27.754777)

SUPPLEMENTARY FIGURE 4

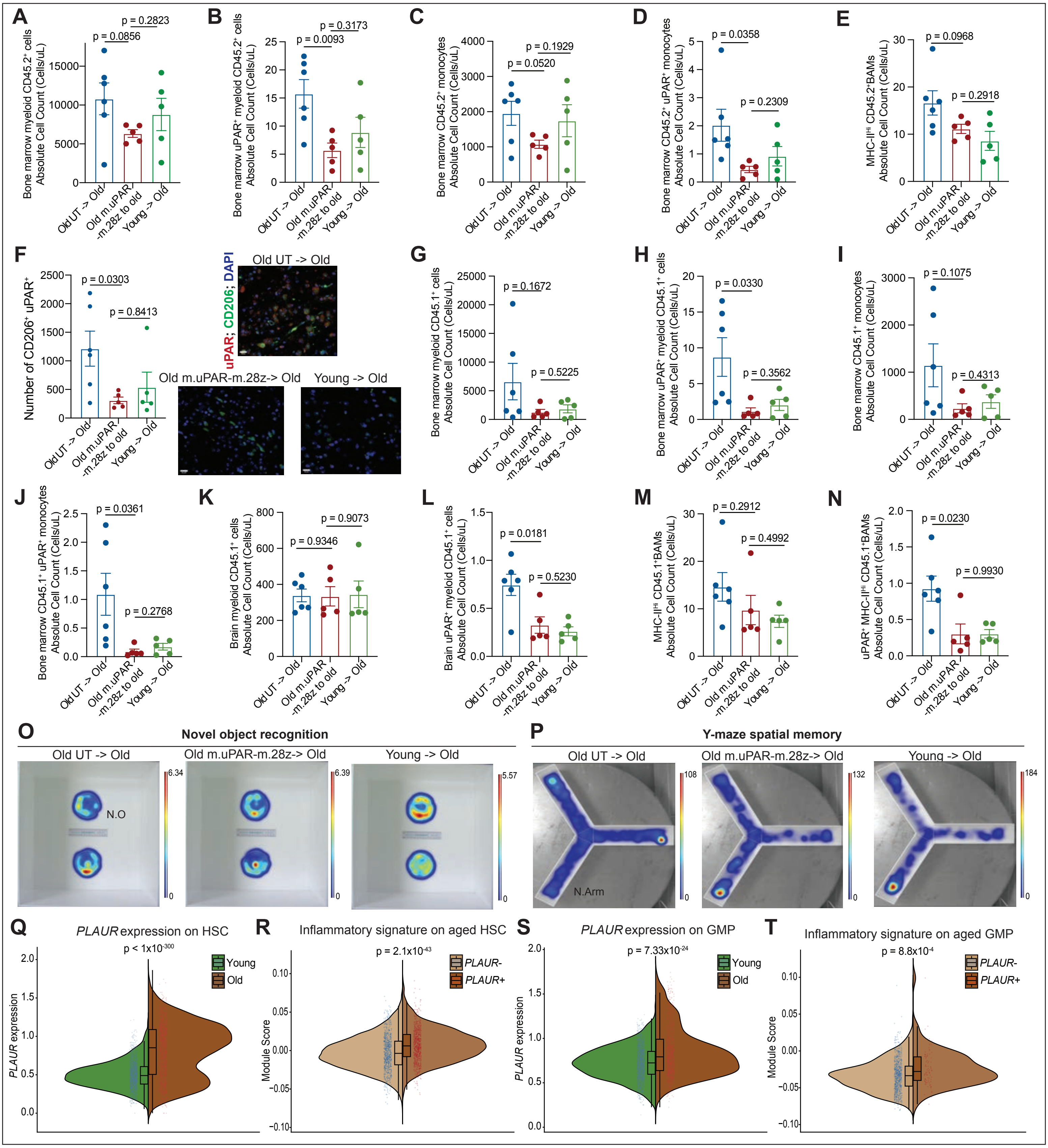

Supplement: Supplementary file 4 [file media-4.pdf]
